# Feasibility of testing and detection of HIV-1 drug resistance in proviral DNA

**DOI:** 10.1186/1471-2334-14-S4-O25

**Published:** 2014-05-29

**Authors:** Clare L Booth, Adele McCormick, Ana Garcia-Diaz, Malcolm Macartney, Michael Youle, Margaret Johnson, Daniel Webster

**Affiliations:** 1 Royal Free London NHS Foundation Trust, London, United Kingdom

## 

In recent years antiretroviral availability and access has become more prevalent in resource poor settings, whilst the availability of access to HIV-1 drug resistance testing remains limited and patients are often started on therapy or switched when failing without a resistance test. Proviral DNA has been proposed as a source of archived resistance mutations. We investigated the feasibility of using proviral DNA to detect previously documented plasma resistance mutations.

Amplification and sequencing of pol from proviral DNA derived from buffy coat (BCL), buffy coat dried on filter paper (BCS) or dried blood spots (DBS) was performed using an in-house assay.

Only 2/20 samples were amplified from long term stored buffy coat (>1 year) compared to at least one prospective sample from 10/20 patients. Six patients had a plasma viral load (PVL) <40 cp/ml at time of sampling, though 4/6 only become undetectable on the most recent sample, 2/4 had been undetectable for 149 and 819 days respectively. The remaining 6 patients had detectable PVL (median 3.1 log10 cp/ml, range: 1.7–6.2 log10 cp/ml). In contrast, 21/28 (75%) of samples which failed to amplify were <40 cp/ml, 2 had become undetectable in the most recent sample and 19 had been undetectable for a median of 577 days (range: 32–2481 days).

6/10 BCL samples showed none of the previously documented resistance for those patients, 3/10 showed some of the previously known mutations and only 1/10 showed all historic resistance, though this patient had only one documented mutation (K103N). For the BCS samples 3/4 showed none of the documented resistance, 1/4 harboured the previously known mutations (M184V, K103N). 3/6 of the DBS had none of the previously documented resistance, 2/6 showed some of the known mutations and 1/6 showed both known resistance mutations (M41L, T215S). In patients where multiple specimen types were amplified, 3/5 showed differences in mutations detected between sample types, with 2/5 correlating but having no mutations detected in any specimens.

In patients with undetectable PVL, particularly those undetectable for long periods, it was difficult to sequence archived DNA. Concordance between different specimen types was variable as was concordance with previously documented mutations. Historic resistance reports remain important in the clinical management of patients on antiretroviral therapy, though proviral DNA testing may be useful in patients where historic reports are not available.

